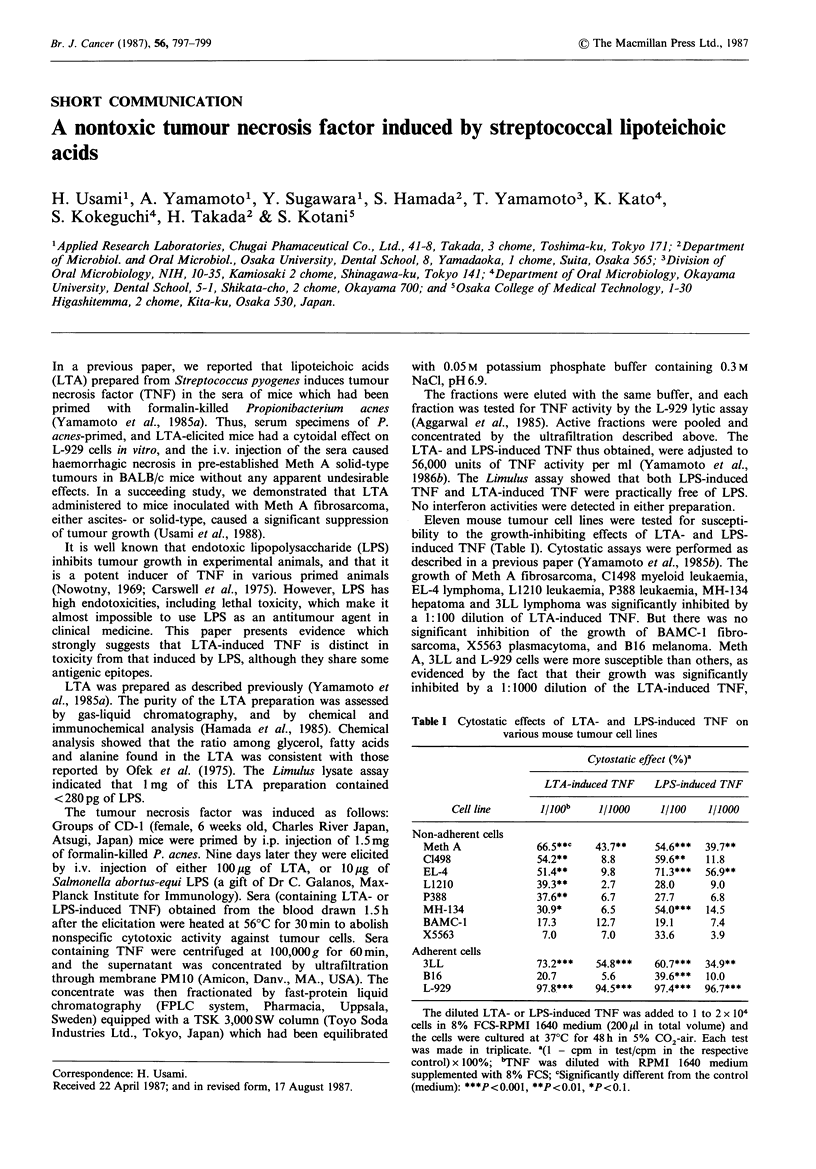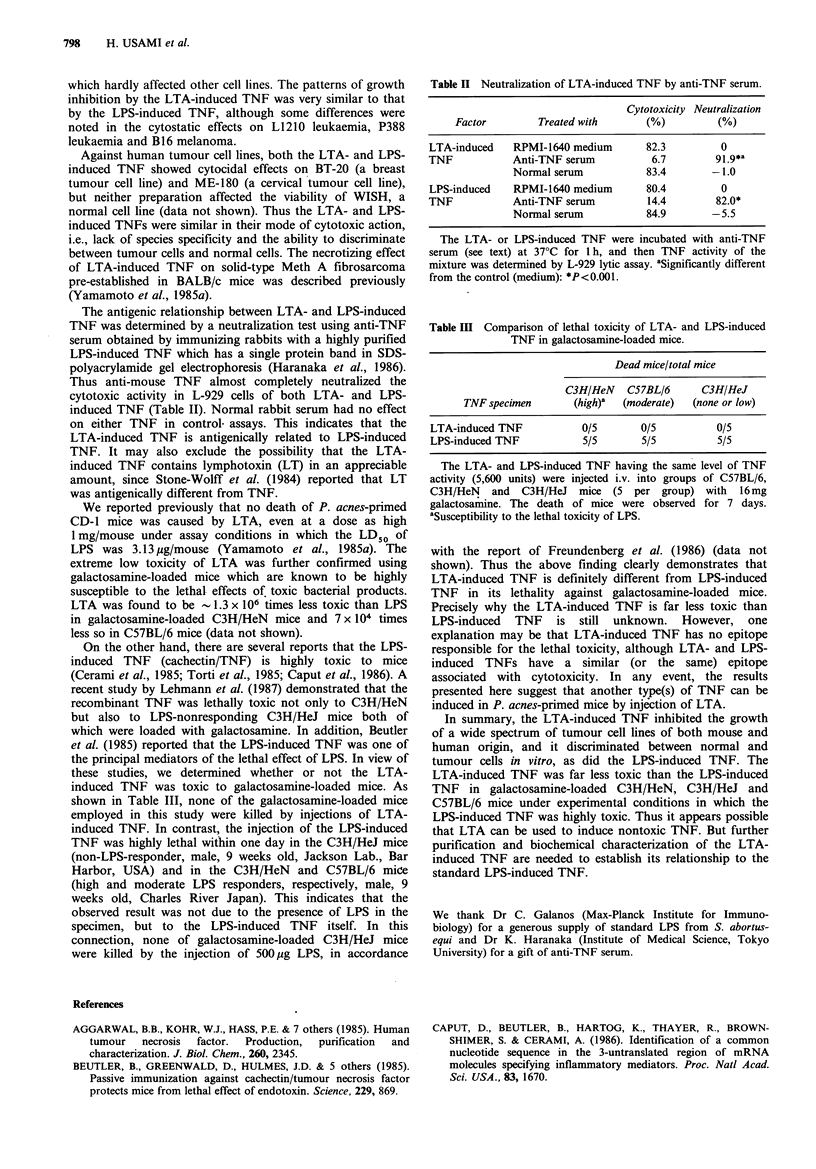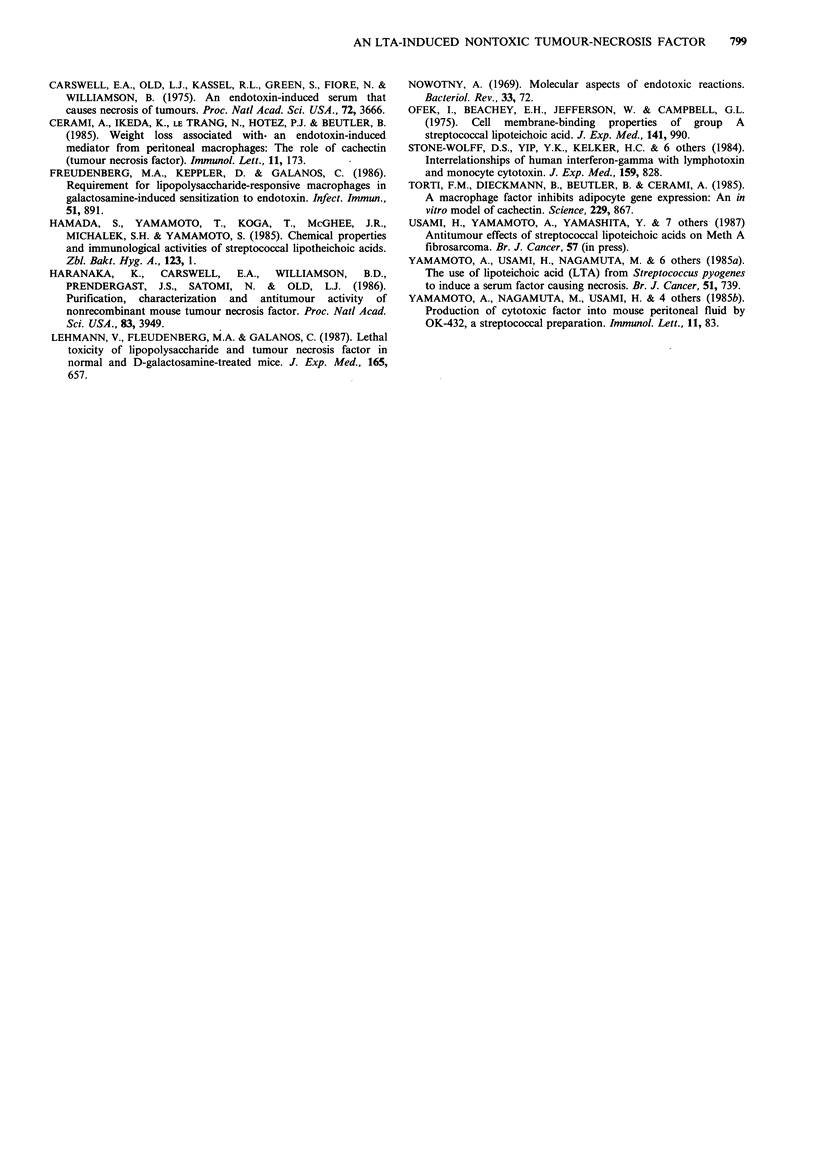# A nontoxic tumour necrosis factor induced by streptococcal lipoteichoic acids.

**DOI:** 10.1038/bjc.1987.292

**Published:** 1987-12

**Authors:** H. Usami, A. Yamamoto, Y. Sugawara, S. Hamada, T. Yamamoto, K. Kato, S. Kokeguchi, H. Takada, S. Kotani

**Affiliations:** Applied Research Laboratories, Chugai Phamaceutical Co., Ltd., Tokyo, Japan.


Br. J. Cancer (1987), 56, 797–799 © The Macmillan Press Ltd., 1987

SHORT COMMUNICATION

### A nontoxic tumour necrosis factor induced by streptococcal lipoteichoic acids

H. Usami[1], A. Yamamoto[1], Y. Sugawara[1], S. Hamada[2], T. Yamamoto[3], K. Kato[4], S. Kokeguchi[4], H. Takada[2] & S. Kotani[5]

[1]*Applied Research Laboratories, Chugai Phamaceutical Co., Ltd., 41-8, Takada, 3 chome, Toshima-ku, Tokyo 171;* [2]*Department of Microbiol. and Oral Microbiol., Osaka University, Dental School, 8, Yamadaoka, 1 chome, Suita, Osaka 565;* [3]*Division of Oral Microbiology, NIH, 10-35, Kamiosaki 2 chome, Shinagawa-ku, Tokyo 141;* [4]*Department of Oral Microbiology, Okayama University, Dental School, 5-1, Shikata-cho, 2 chome, Okayama 700; and* [5]*Osaka College of Medical Technology, 1-30 Higashitemma, 2 chome, Kita-ku, Osaka 530, Japan.*

In a previous paper, we reported that lipoteichoic acids (LTA) prepared from *Streptococcus pyogenes* induces tumour necrosis factor (TNF) in the sera of mice which had been primed with formalin-killed *Propionibacterium acnes* (Yamamoto *et al.*, 1985*a*). Thus, serum specimens of *P. acnes*-primed, and LTA-elicited mice had a cytoidal effect on L-929 cells *in vitro*, and the i.v. injection of the sera caused haemorrhagic necrosis in pre-established Meth A solid-type tumours in BALB/c mice without any apparent undesirable effects. In a succeeding study, we demonstrated that LTA administered to mice inoculated with Meth A fibrosarcoma, either ascites- or solid-type, caused a significant suppression of tumour growth (Usami *et al.*, 1988).

It is well known that endotoxic lipopolysaccharide (LPS) inhibits tumour growth in experimental animals, and that it is a potent inducer of TNF in various primed animals (Nowotny, 1969; Carswell *et al.*, 1975). However, LPS has high endotoxicities, including lethal toxicity, which make it almost impossible to use LPS as an antitumour agent in clinical medicine. This paper presents evidence which strongly suggests that LTA-induced TNF is distinct in toxicity from that induced by LPS, although they share some antigenic epitopes.

LTA was prepared as described previously (Yamamoto *et al.*, 1985*a*). The purity of the LTA preparation was assessed by gas-liquid chromatography, and by chemical and immunochemical analysis (Hamada *et al.*, 1985). Chemical analysis showed that the ratio among glycerol, fatty acids and alanine found in the LTA was consistent with those reported by Ofek *et al.* (1975). The *Limulus* lysate assay indicated that 1 mg of this LTA preparation contained <280 pg of LPS.

The tumour necrosis factor was induced as follows: Groups of CD-1 (female, 6 weeks old, Charles River Japan, Atsugi, Japan) mice were primed by i.p. injection of 1.5 mg of formalin-killed *P. acnes.* Nine days later they were elicited by i.v. injection of either 100 μg of LTA, or 10 μg of *Salmonella abortus-equi* LPS (a gift of Dr C. Galanos, Max-Planck Institute for Immunology). Sera (containing LTA- or LPS-induced TNF) obtained from the blood drawn 1.5 h after the elicitation were heated at 56°C for 30 min to abolish nonspecific cytotoxic activity against tumour cells. Sera containing TNF were centrifuged at 100,000 *g* for 60 min, and the supernatant was concentrated by ultrafiltration through membrane PM10 (Amicon, Danv., MA., USA). The concentrate was then fractionated by fast-protein liquid chromatography (FPLC system, Pharmacia, Uppsala, Sweden) equipped with a TSK 3,000 SW column (Toyo Soda Industries Ltd., Tokyo, Japan) which had been equilibrated with 0.05 M potassium phosphate buffer containing 0.3 M NaCl, pH 6.9.

The fractions were eluted with the same buffer, and each fraction was tested for TNF activity by the L-929 lytic assay (Aggarwal *et al.*, 1985). Active fractions were pooled and concentrated by the ultrafiltration described above. The LTA- and LPS-induced TNF thus obtained, were adjusted to 56,000 units of TNF activity per ml (Yamamoto *et al.*, 1986*b*). The *Limulus* assay showed that both LPS-induced TNF and LTA-induced TNF were practically free of LPS. No interferon activities were detected in either preparation.

Eleven mouse tumour cell lines were tested for susceptibility to the growth-inhibiting effects of LTA- and LPS-induced TNF (Table I). Cytostatic assays were performed as described in a previous paper (Yamamoto *et al.*, 1985*b*). The growth of Meth A fibrosarcoma, C1498 myeloid leukaemia, EL-4 lymphoma, L1210 leukaemia, P388 leukaemia, MH-134 hepatoma and 3LL lymphoma was significantly inhibited by a 1:100 dilution of LTA-induced TNF. But there was no significant inhibition of the growth of BAMC-1 fibrosarcoma, X5563 plasmacytoma, and B16 melanoma. Meth A, 3LL and L-929 cells were more susceptible than others, as evidenced by the fact that their growth was significantly inhibited by a 1:1000 dilution of the LTA-induced TNF,

**Table I** Cytostatic effects of LTA- and LPS-induced TNF on various mouse tumour cell lines

| | *Cytostatic effect* (%)[a] | | | |
|---|---|---|---|---|
| | *LTA-induced TNF* | | *LPS-induced TNF* | |
| *Cell line* | *1/100*[b] | *1/1000* | *1/100* | *1/1000* |
| Non-adherent cells | | | | |
| Meth A | 66.5***[c] | 43.7** | 54.6*** | 39.7** |
| C1498 | 54.2** | 8.8 | 59.6** | 11.8 |
| EL-4 | 51.4** | 9.8 | 71.3*** | 56.9** |
| L1210 | 39.3** | 2.7 | 28.0 | 9.0 |
| P388 | 37.6** | 6.7 | 27.7 | 6.8 |
| MH-134 | 30.9* | 6.5 | 54.0*** | 14.5 |
| BAMC-1 | 17.3 | 12.7 | 19.1 | 7.4 |
| X5563 | 7.0 | 7.0 | 33.6 | 3.9 |
| Adherent cells | | | | |
| 3LL | 73.2*** | 54.8*** | 60.7*** | 34.9** |
| B16 | 20.7 | 5.6 | 39.6*** | 10.0 |
| L-929 | 97.8*** | 94.5*** | 97.4*** | 96.7*** |

The diluted LTA- or LPS-induced TNF was added to 1 to $2 \times 10^4$ cells in 8% FCS-RPMI 1640 medium (200 μl in total volume) and the cells were cultured at 37°C for 48 h in 5% $CO_2$-air. Each test was made in triplicate. [a](1 – cpm in test/cpm in the respective control) × 100%; [b]TNF was diluted with RPMI 1640 medium supplemented with 8% FCS; [c]Significantly different from the control (medium): ***$P<0.001$, **$P<0.01$, *$P<0.1$.

Correspondence: H. Usami.
Received 22 April 1987; and in revised form, 17 August 1987.

which hardly affected other cell lines. The patterns of growth inhibition by the LTA-induced TNF was very similar to that by the LPS-induced TNF, although some differences were noted in the cytostatic effects on L1210 leukaemia, P388 leukaemia and B16 melanoma.

Against human tumour cell lines, both the LTA- and LPS-induced TNF showed cytocidal effects on BT-20 (a breast tumour cell line) and ME-180 (a cervical tumour cell line), but neither preparation affected the viability of WISH, a normal cell line (data not shown). Thus the LTA- and LPS-induced TNFs were similar in their mode of cytotoxic action, i.e., lack of species specificity and the ability to discriminate between tumour cells and normal cells. The necrotizing effect of LTA-induced TNF on solid-type Meth A fibrosarcoma pre-established in BALB/c mice was described previously (Yamamoto *et al.*, 1985*a*).

The antigenic relationship between LTA- and LPS-induced TNF was determined by a neutralization test using anti-TNF serum obtained by immunizing rabbits with a highly purified LPS-induced TNF which has a single protein band in SDS-polyacrylamide gel electrophoresis (Haranaka *et al.*, 1986). Thus anti-mouse TNF almost completely neutralized the cytotoxic activity in L-929 cells of both LTA- and LPS-induced TNF (Table II). Normal rabbit serum had no effect on either TNF in control assays. This indicates that the LTA-induced TNF is antigenically related to LPS-induced TNF. It may also exclude the possibility that the LTA-induced TNF contains lymphotoxin (LT) in an appreciable amount, since Stone-Wolff *et al.* (1984) reported that LT was antigenically different from TNF.

We reported previously that no death of *P. acnes*-primed CD-1 mice was caused by LTA, even at a dose as high 1 mg/mouse under assay conditions in which the $LD_{50}$ of LPS was 3.13 μg/mouse (Yamamoto *et al.*, 1985*a*). The extreme low toxicity of LTA was further confirmed using galactosamine-loaded mice which are known to be highly susceptible to the lethal effects of toxic bacterial products. LTA was found to be $\sim 1.3 \times 10^6$ times less toxic than LPS in galactosamine-loaded C3H/HeN mice and $7 \times 10^4$ times less so in C57BL/6 mice (data not shown).

On the other hand, there are several reports that the LPS-induced TNF (cachectin/TNF) is highly toxic to mice (Cerami *et al.*, 1985; Torti *et al.*, 1985; Caput *et al.*, 1986). A recent study by Lehmann *et al.* (1987) demonstrated that the recombinant TNF was lethally toxic not only to C3H/HeN but also to LPS-nonresponding C3H/HeJ mice both of which were loaded with galactosamine. In addition, Beutler *et al.* (1985) reported that the LPS-induced TNF was one of the principal mediators of the lethal effect of LPS. In view of these studies, we determined whether or not the LTA-induced TNF was toxic to galactosamine-loaded mice. As shown in Table III, none of the galactosamine-loaded mice employed in this study were killed by injections of LTA-induced TNF. In contrast, the injection of the LPS-induced TNF was highly lethal within one day in the C3H/HeJ mice (non-LPS-responder, male, 9 weeks old, Jackson Lab., Bar Harbor, USA) and in the C3H/HeN and C57BL/6 mice (high and moderate LPS responders, respectively, male, 9 weeks old, Charles River Japan). This indicates that the observed result was not due to the presence of LPS in the specimen, but to the LPS-induced TNF itself. In this connection, none of galactosamine-loaded C3H/HeJ mice were killed by the injection of 500 μg LPS, in accordance with the report of Freundenberg *et al.* (1986) (data not shown). Thus the above finding clearly demonstrates that LTA-induced TNF is definitely different from LPS-induced TNF in its lethality against galactosamine-loaded mice. Precisely why the LTA-induced TNF is far less toxic than LPS-induced TNF is still unknown. However, one explanation may be that LTA-induced TNF has no epitope responsible for the lethal toxicity, although LTA- and LPS-induced TNFs have a similar (or the same) epitope associated with cytotoxicity. In any event, the results presented here suggest that another type(s) of TNF can be induced in *P. acnes*-primed mice by injection of LTA.

In summary, the LTA-induced TNF inhibited the growth of a wide spectrum of tumour cell lines of both mouse and human origin, and it discriminated between normal and tumour cells *in vitro*, as did the LPS-induced TNF. The LTA-induced TNF was far less toxic than the LPS-induced TNF in galactosamine-loaded C3H/HeN, C3H/HeJ and C57BL/6 mice under experimental conditions in which the LPS-induced TNF was highly toxic. Thus it appears possible that LTA can be used to induce nontoxic TNF. But further purification and biochemical characterization of the LTA-induced TNF are needed to establish its relationship to the standard LPS-induced TNF.

**Table II** Neutralization of LTA-induced TNF by anti-TNF serum.

| *Factor* | *Treated with* | *Cytotoxicity* (%) | *Neutralization* (%) |
|---|---|---|---|
| LTA-induced TNF | RPMI-1640 medium | 82.3 | 0 |
| | Anti-TNF serum | 6.7 | 91.9*[a] |
| | Normal serum | 83.4 | −1.0 |
| LPS-induced TNF | RPMI-1640 medium | 80.4 | 0 |
| | Anti-TNF serum | 14.4 | 82.0* |
| | Normal serum | 84.9 | −5.5 |

The LTA- or LPS-induced TNF were incubated with anti-TNF serum (see text) at 37°C for 1 h, and then TNF activity of the mixture was determined by L-929 lytic assay. [a]Significantly different from the control (medium): $*P < 0.001$.

**Table III** Comparison of lethal toxicity of LTA- and LPS-induced TNF in galactosamine-loaded mice.

| | *Dead mice/total mice* | | |
|---|---|---|---|
| *TNF specimen* | *C3H/HeN (high)*[a] | *C57BL/6 (moderate)* | *C3H/HeJ (none or low)* |
| LTA-induced TNF | 0/5 | 0/5 | 0/5 |
| LPS-induced TNF | 5/5 | 5/5 | 5/5 |

The LTA- and LPS-induced TNF having the same level of TNF activity (5,600 units) were injected i.v. into groups of C57BL/6, C3H/HeN and C3H/HeJ mice (5 per group) with 16 mg galactosamine. The death of mice were observed for 7 days. [a]Susceptibility to the lethal toxicity of LPS.

We thank Dr C. Galanos (Max-Planck Institute for Immunobiology) for a generous supply of standard LPS from *S. abortus-equi* and Dr K. Haranaka (Institute of Medical Science, Tokyo University) for a gift of anti-TNF serum.

### References

AGGARWAL, B.B., KOHR, W.J., HASS, P.E. & 7 others (1985). Human tumour necrosis factor. Production, purification and characterization. *J. Biol. Chem.*, **260**, 2345.

BEUTLER, B., GREENWALD, D., HULMES, J.D. & 5 others (1985). Passive immunization against cachectin/tumour necrosis factor protects mice from lethal effect of endotoxin. *Science*, **229**, 869.

CAPUT, D., BEUTLER, B., HARTOG, K., THAYER, R., BROWN-SHIMER, S. & CERAMI, A. (1986). Identification of a common nucleotide sequence in the 3-untranslated region of mRNA molecules specifying inflammatory mediators. *Proc. Natl Acad. Sci. USA.*, **83**, 1670.

CARSWELL, E.A., OLD, L.J., KASSEL, R.L., GREEN, S., FIORE, N. & WILLIAMSON, B. (1975). An endotoxin-induced serum that causes necrosis of tumours. *Proc. Natl Acad. Sci. USA.*, **72,** 3666.

CERAMI, A., IKEDA, K., LE TRANG, N., HOTEZ, P.J. & BEUTLER, B. (1985). Weight loss associated with an endotoxin-induced mediator from peritoneal macrophages: The role of cachectin (tumour necrosis factor). *Immunol. Lett.*, **11,** 173.

FREUDENBERG, M.A., KEPPLER, D. & GALANOS, C. (1986). Requirement for lipopolysaccharide-responsive macrophages in galactosamine-induced sensitization to endotoxin. *Infect. Immun.*, **51,** 891.

HAMADA, S., YAMAMOTO, T., KOGA, T., MCGHEE, J.R., MICHALEK, S.H. & YAMAMOTO, S. (1985). Chemical properties and immunological activities of streptococcal lipotheichoic acids. *Zbl. Bakt. Hyg. A.*, **123,** 1.

HARANAKA, K., CARSWELL, E.A., WILLIAMSON, B.D., PRENDERGAST, J.S., SATOMI, N. & OLD, L.J. (1986). Purification, characterization and antitumour activity of nonrecombinant mouse tumour necrosis factor. *Proc. Natl Acad. Sci. USA.*, **83,** 3949.

LEHMANN, V., FLEUDENBERG, M.A. & GALANOS, C. (1987). Lethal toxicity of lipopolysaccharide and tumour necrosis factor in normal and D-galactosamine-treated mice. *J. Exp. Med.*, **165,** 657.

NOWOTNY, A. (1969). Molecular aspects of endotoxic reactions. *Bacteriol. Rev.*, **33,** 72.

OFEK, I., BEACHEY, E.H., JEFFERSON, W. & CAMPBELL, G.L. (1975). Cell membrane-binding properties of group A streptococcal lipoteichoic acid. *J. Exp. Med.*, **141,** 990.

STONE-WOLFF, D.S., YIP, Y.K., KELKER, H.C. & 6 others (1984). Interrelationships of human interferon-gamma with lymphotoxin and monocyte cytotoxin. *J. Exp. Med.*, **159,** 828.

TORTI, F.M., DIECKMANN, B., BEUTLER, B. & CERAMI, A. (1985). A macrophage factor inhibits adipocyte gene expression: An *in vitro* model of cachectin. *Science*, **229,** 867.

USAMI, H., YAMAMOTO, A., YAMASHITA, Y. & 7 others (1987) Antitumour effects of streptococcal lipoteichoic acids on Meth A fibrosarcoma. *Br. J. Cancer*, **57** (in press).

YAMAMOTO, A., USAMI, H., NAGAMUTA, M. & 6 others (1985*a*). The use of lipoteichoic acid (LTA) from *Streptococcus pyogenes* to induce a serum factor causing necrosis. *Br. J. Cancer*, **51,** 739.

YAMAMOTO, A., NAGAMUTA, M., USAMI, H. & 4 others (1985*b*). Production of cytotoxic factor into mouse peritoneal fluid by OK-432, a streptococcal preparation. *Immunol. Lett.*, **11,** 83.